# Apocrine secretion in the salivary glands of *Drosophilidae* and other dipterans is evolutionarily conserved

**DOI:** 10.3389/fcell.2022.1088055

**Published:** 2023-01-13

**Authors:** Klaudia Babišová, Lucia Mentelová, Terézia Klaudia Geisseová, Denisa Beňová-Liszeková, Milan Beňo, Bruce A. Chase, Robert Farkaš

**Affiliations:** ^1^ Laboratory of Developmental Genetics, Institute of Experimental Endocrinology, Biomedical Research Center v.v.i., Slovak Academy of Sciences, Bratislava, Slovakia; ^2^ Department of Genetics, Comenius University, Bratislava, Slovakia; ^3^ Department of Biology, University of Nebraska, Omaha, NE, United States

**Keywords:** apocrine secretion, prepupal salivary glands, *Drosophila* species, evolutionarily conserved function, exocytotic salivary gland glue secretion

## Abstract

Apocrine secretion is a transport and secretory mechanism that remains only partially characterized, even though it is evolutionarily conserved among all metazoans, including humans. The excellent genetic model organism *Drosophila melanogaster* holds promise for elucidating the molecular mechanisms regulating this fundamental metazoan process. Two prerequisites for such investigations are to clearly define an experimental system to investigate apocrine secretion and to understand the evolutionarily and functional contexts in which apocrine secretion arose in that system. To this end, we recently demonstrated that, in *D. melanogaster*, the prepupal salivary glands utilize apocrine secretion prior to pupation to deliver innate immune and defense components to the exuvial fluid that lies between the metamorphosing pupae and its chitinous case. This finding provided a unique opportunity to appraise how this novel non-canonical and non-vesicular transport and secretory mechanism is employed in different developmental and evolutionary contexts. Here we demonstrate that this apocrine secretion, which is mechanistically and temporarily separated from the exocytotic mechanism used to produce the massive salivary glue secretion (Sgs), is shared across *Drosophilidae* and two unrelated dipteran species. Screening more than 30 species of *Drosophila* from divergent habitats across the globe revealed that apocrine secretion is a widespread and evolutionarily conserved cellular mechanism used to produce exuvial fluid. Species with longer larval and prepupal development than *D*. *melanogaster* activate apocrine secretion later, while smaller and more rapidly developing species activate it earlier. In some species, apocrine secretion occurs after the secretory material is first concentrated in cytoplasmic structures of unknown origin that we name “collectors.” Strikingly, in contrast to the widespread use of apocrine secretion to provide exuvial fluid, not all species use exocytosis to produce the viscid salivary glue secretion that is seen in *D. melanogaster*. Thus, apocrine secretion is the conserved mechanism used to realize the major function of the salivary gland in fruitflies and related species: it produces the pupal exuvial fluid that provides an active defense against microbial invasion during pupal metamorphosis.

## Introduction

Apocrine secretion was among the earliest secretory mechanisms to be recognized, however, still it remains enigmatic. The first paper on an apocrine secretory organ is that of Harder, (1694) who described a special lachrymal gland in rodents. Some 140 years later, the human sweat gland, one of the most intensely studied apocrine organ, was discovered by Purkyně (also known as Purkinje) (Purkinje, 1833a; Purkinje, 1833b) and further described in detail by his student Wendt (1833, 1834). Later Velpeau (1839) and Verneuil (1854) independently described a chronic acneiform disease of the cutaneous sweat (apocrine) glands that later was named hidradenitis suppurativa (HS) (Richter, 1932; Lasko et al., 2008). Ranvier (1879) was the first to distinguish “holocrine” secretion in the sebaceous gland from “eccrine/merocrine” secretion in the sweat glands. But it was not until 1917 and 1921 when Schiefferdecker, based on Ranvier’s observations, suggested that the sweat gland cells be classified functionally according to how they secreted their contents, by an eccrine/merocrine, apocrine or holocrine mechanism (Schiefferdecker, 1917; 1921). This was a breakthrough contribution. It established a clear functional definition of three substantially different categories of secretion based on the mechanism underlying the externalization of cellular materials. Nonetheless, for almost a century afterward, the classic “textbook” description of apocrine secretion was framed in terms of observations on mammary glands: lipid vacuoles or droplets arising in the cytoplasm bulge from a cell’s apical pole as large spherical protrusions. Mechanistic details were unclear: some authors consider that apocrine secretion in the Harderian or mammary gland involved only the release of lipids, whereas proteins (e.g., casein vesicles) were released by exocytosis (Bubenik et al., 1976; Brownscheidle and Niewenhuis, 1978; Dylewski and Keenan, 1983; Kralj and Pipan, 1992). However, the discovery of massive apocrine secretion in the prepupal salivary glands of *Drosophila*, as successful model organism, allowed us reappraise our understanding of apocrine secretion using insights specific and unique to this model (Farkaš et al., 2014; Farkaš, 2015; Farkaš, 2016).

The larval salivary glands (SGs) of *Drosophila melanogaster* are a single layer of epithelial cells that form an unbranched, tubular secretory tissue of ectodermal origin (Skaer, 1993; Campos-Ortega and Hartenstein, 1997; Bradley et al., 2001; Vining et al., 2005; Kerman et al., 2006; Farkaš, 2016). For over six decades, the only well documented function of the mature SGs was the production of abundant mucinous secretory granules during the second half of last instar, which are released by a typical exocytotic mechanism prior to pupariation to serve as a glue that affixes a freshly formed puparia to a substrate (Fraenkel and Brookes, 1953; Lehmann, 1996; Farkaš, 2016). Although it has not been formally demonstrated by screening species across the cyclorrhaphous dipterans (infraorder *Muscomorpha*), it has long been assumed that the universal and major evolutionary function of the larval SGs in these insects is to produce this mucinous glue (Fraenkel and Brookes, 1953).

Hitherto, we have described apocrine secretion in the *Drosophila* prepupal SGs (Farkaš et al., 2014). In contrast to the well defined mechanism of exocytosis (Südhof, 2004; Südhof and Rothman, 2009; Anantharam et al., 2010; Murray and Stow, 2014; Anantharam and Kreutzberger, 2019; Maj et al., 2019), apocrine secretion is a non-vesicular and non-canonical transport and secretory pathway that entails the loss of part of the cytoplasm during which homotypic membrane fusion is not required. Apocrine secretion involves apical protrusions often associated with cytoplasmic fragments inside a secretory lumen (Farkaš, 2015). As we postulated previously, in its most intense phase apocrine secretion is accompanied by the release of large fragments of cytoplasm and other cellular structures including entire organelles including microsomes, mitochondria, Golgi, and portions of the ER (Farkaš et al., 2014). It occurs separately from, i.e., earlier than, apoptotic programmed cell death (Farkaš et al., 2014). Proteomic analyses revealed that apocrine secretory material contains hundreds to thousands of microsomal, mitochondrial, ribosomal, membranous, cytoskeletal, nuclear, and even nucleolar proteins. Strikingly, although many nuclear proteins, including transcription factors, chromatin components and remodeling factors are released, the nuclear DNA itself remains intact (Farkaš et al., 2014). Interspecies comparison of proteomes from *Drosophila* and several human apocrine glands found that the distribution of the numerous protein components of apocrine secretion among ontological categories is almost identical, regardless of the evolutionary distance and anatomical location of the apocrine gland (Farkaš, 2015; Farkaš, 2016, Farkaš et al., 2015). An additional shared feature of apocrine secretion across species appears to be that it is common to many, if not all, barrier epithelial tissues. In humans these include skin derivatives and the epididymis, and apocrine secretion is implicated also in lung/bronchi and intestinal epithelium. Therefore, elucidating the molecular-genetic mechanism of apocrine secretion used by all animals should be possible using the unique genetic tools available in *Drosophila*, just as exocytosis was deciphered using Drosophila and yeast as working model organism (Südhof, 2004; Snyder et al., 2006; Deak et al., 2008; Südhof and Rothman, 2009; Wu et al., 2014).

We recently demonstrated that apocrine secretion in the prepupal SGs of *D. melanogaster* produces the exuvial fluid that lies between the pupae and its chitinous case (Beňová-Liszeková et al., 2021). Consistent with the secretion providing an essential function and occurring earlier than programmed cell death (Farkaš et al., 2014), we demonstrated that the secretion contains numerous antimicrobial and antibacterial factors: the secretion functions to provide an innate-immunity barrier to protect the metamorphosing pupae. In *D. melanogaster,* anatomical examination revealed that in the late prepupa, the pair of SGs share a single common salivary duct that only connects to the floor of the pharynx. This connection delivers the apocrine secretion into the periexuvial cavity instead of the alimentary tract, which becomes histolyzed shortly after pupariation. To verify the release of the apocrine secretion into this space, we had followed the fate of a Green-Fluorescent-Protein (GFP) marker strongly expressed in the late larval and early prepupal salivary glands. We found components of apocrine secretion including heterologous GFP in exuvial fluid and verified their presence by mass spectrometry, confirming that the apocrine secretion from prepupal SGs is identical to exuvial fluid.

A fundamental unanswered question is whether apocrine secretion in the SGs of *D. melanogaster* is a species-specific phenomenon. Alternatively, it could be restricted to other, perhaps closely related species, or occur in many or even all members of the *Drosophila* genus. This is a significant question as the different organs that can utilize an apocrine mechanism for secretion in vertebrates do not do so across all vertebrate species. Variation across vertebrates in the use of an apocrine mechanism for secretion has been found in the sweat glands, middle-ear glands, lacrimal glands, the bronchoalveolar epithelium, the epididymis, the choroid plexus, the parathyroid gland, the anterior pituitary gland, the coagulating gland, the SGs, and the infraorbital gland (Smith and Hearn, 1979; Agnew et al., 1980; Kurosumi et al., 1981; Schwarz et al., 1988; Morales and Cavicchia, 1991; Atoji et al., 1993; Atoji et al., 1998; Payne, 1994; Gesase and Satoh, 2003; Cleveland et al., 2012; Farkaš, 2015), which are mostly studied in mammals and particularly in humans. Here, we present data from a pantheon of 32 *Drosophila* and two more distant *Diptera* species originating from diverse locations across the globe, including the Neotropical, Palearctic, Indomalaysian, Afrotropic, Australasian, and Nearctic regions, and covering rain forest, desert, temperate-montane forest and many other habitats. We demonstrate that apocrine secretion by the prepupal SGs is widespread and evolutionarily conserved among all of the species from these very divergent habitats and geographical regions. Strikingly, the massive exocytotic secretion that is typical for *D. melanogaster*, which occurs prior to puparization and is associated with the production of salivary gland glue (Sgs), is not conserved in all of the species we examined. This indicates that the major secretory function of this organ in fruitflies is connected with active defense against microbial invasion *via* the production and subsequent apocrine secretion of pupal exuvial fluid.

We find that, while there are some species-specific differences in the intensity, appearance and speed of apocrine secretion, there are no clear-cut correlations between these aspects of apocrine secretion and a prior exocytotic release of secretory Sgs-glue, the number of polytene cells per gland, or the geographic or climatic region. Apocrine secretion in species with longer larval and prepupal development is later than that in *D*. *melanogaster*, while it is earlier in several smaller and more rapidly developing species that pupate earlier. In a few species, the secretory material is concentrated in cytoplasmic structures of unknown origin before apocrine secretion occurs, which we name sacculae or “collectors.”

## Materials and methods

### Fly species and culturing

Most of the fly species we examined were cultured in 50 ml vials or 200 ml bottles at 23°C on agar-yeast-cornmeal-molasses medium (Ashburner and Thompson, 1978; Ransom, 1982) with the addition of methylparaben to prevent molds. Several species required growth on species-specific media which we prepared as described by Markow and O’Grady, (2005). We used observations carried out on the last (3rd) instar larvae of wild-type *D. melanogaster* (Meigen) *Oregon R,* originally obtained from the Umea *Drosophila* Stock Centre, Umea Sweden, as a standard reference control (Lindsley and Zimm, 1992). Other *Drosophila* species we examined are listed below.

The species *D*. *willistoni*, *D*. *virilis*, *D*. *atripex*, *D*. *yakuba*, *D*. *pallidosa*, *D*. *ananassae*, *D*. *mauritiana*, *D*. *pseudoananassae*, *D*. *bipectinata*, and *D*. *parabipectinata* were generous gift of Gábor Csórda and Victor Honti of the Biological Research Centre, Hungarian Academy of Sciences, Szeged, Hungary. The *D*. *simulans* was a gift of Christian Schlötterer, Institute of Population Genetics, University of Veterinary Medicine, Vienna. The species *D*. *mojavensis*, *D*. *sulfurigaster*, *D*. *subobscura*, *D*. *immigans*, *D*. *birchii*, and *D*. *equinoxialis* were generously provided by Johannes Overgaard and Heidi MacLean of Aarhus University, Denmark. All these species were reared on the same agar-yeast-cornmeal-molasses diet as *D*. *melanogaster*. *D*. *pseudoobscura*, *D*. *persimilis*, *D*. *erecta*, *D*. *sechellia*, and *D*. *eugracilis* were generous gift of Élio Sucena, Gulbenkian Institute of Science, Oeiras, Portugal. Both, *D*. *montana* and *Chymomyza costata* were obtained from Vladimír Košťál, Institute of Entomology, Czech Academy of Sciences, České Budějovice, Czech Republic. *D*. *montana* was reared at 20°C–22°C on agar-cornmeal-sugar diet without yeast and propionic acid, and *C*. *costata* (wild type *Sapporo* strain) were cultured on an artificial acid-free barley malt-based diet of Lakovaara (1969) under a constant temperature of 18°C–20°C and a long-day photoperiod (22 h light: 2 h darkness) to prevent diapause as described by Košťál et al. (1998). Under these conditions, mature 3rd instar larvae of *C*. *costata* leave food media to pupariate on a piece of tuft-fan folded filter paper inserted into the media during the 2 h dark period, and tend to pupariate relatively quickly—within 1–2 h. That is, their wandering period was either very short or absent. If a folded filter paper was not inserted in the food, these larvae pupariated on the wall of the glass vial in the vicinity of the food or in it and did not climb far from the food medium.

All other species were obtained from San Diego *Drosophila* Species Stock Center (now *Drosophila* Species Stock Center at Cornell University, Ithaca, New York), and reared according to protocols described in Markow and O’Grady, (2005). The mushroom, banana-opuntia, Saguaro-potato and Wheeler-Clayton food for some species was prepared according to the recipes described at *Drosophila* Species Stock Center (https://stockcenter.ucsd.edu, now http://blogs.cornell.edu/drosophila/). Agar and methylparaben came from Sigma, brewer yeasts and barley malt were from Heineken brewery (Hurbanovo, Slovakia), yeast extract was from Difco (Becton-Dickinson), propionic acid from Fisher Scientific. All other ingredients (cornmeal, portabella mushroom powder, blended banana, opuntia cactus powder, Karo syrup) were from local Slovak or United States food stores (Gerber, Karo, Earth’s Best, Kellogg’s, Hoosier Hill Farm, Salvia Paradise, SunFood VM, StarWest Botanicals, Nubeleaf and CountryLifeBio).

Larvae of *Lucilia cuprina* were reared according to Greenberg and George (1985) and fed on ground beef liver mixed with bran. The same liver was used also as a medium for oviposition. Last instar larvae that ceased feeding were transferred to fresh cages to await pupariation, and freshly pupariated animals were collected, and counted as 0-h old.

The black soldier flies (*Hermetia illucens*, Linnaeus, 1758) were reared according to Sheppard et al. (2002) and Tomberlin et al. (2002) as modified by Holmes et al. (2013). They were maintained in complete dark at 24°C ± 1°C with 70%–85% relative humidity and fed *ad libitum* discarded organic food that included potatoes and other vegetables, fruits, bread, and beef, chicken and pork meat. *H. illucens* has six larval instars that take 25–35 days, a prepupal stage lasting 7–10 days, and a pupal stage lasting 14 days (Barros et al., 2019; Bonelli et al., 2020). The live weight of the last instar larva of *Hermetia* is 115 to 125-fold heavier than that of *D*. *melanogaster*. In contrast to more well studied cyclorrhaphous dipterans, the wandering stage of their last instar larvae is extended, and persists during the entire prepupal period. While both the larvae and prepupae are negatively phototropic, the prepupae are especially sensitive to light. For these analyses, salivary glands were dissected from wandering 6th instar larvae, prepupal animals on a daily basis and 1 to 3 days-old pupae.

### Identification of the pupation time and the time of apocrine secretion in non-*melanogaster* species

In *D*. *melanogaster*, the time of apocrine secretion is 8–10 h after puparium formation (APF) while pupation is 12.5–13 h APF (Farkaš et al., 2014). We initially expected to be able to extrapolate a time scale for apocrine secretion from this information in other species. Except for some unverified information from *D*. *hydei* or *D*. *simulans*, however, very little information was available on key metamorphic events, including pupation in the species used in this study. To fill this gap, each species was maintained at a particular temperature (20°C–25°C ± 1°C, as detailed above) and animals at the white puparium stage (0 h prepupa) carefully selected. The freshly formed prepupae were briefly washed under a gentle stream of water to remove food remnants from their body surface and placed on a clean microscopic glass slide inside of a humid chamber. Two or three rows, each with 10 animals were made, and the animals observed under a Wild/Leica MZ-9^.5^ stereomicroscope with transmitted illumination, so that the contours of the animal remained visible even after the darkening of the tanned prepupal case. Observations on the animals were recorded every hour during the first 6 h, and then every 15 min until the sharp contours of the everted pupal head and expelled larval mouth hooks could be observed, as these together identify the beginning of the pupal stage. The times of pupation and apocrine secretion for a species are the range of times seen in three sets of observations, each having a minimum of 30 animals. The data are presented in Table 1.

**TABLE 1:** Summary data on the activities and developmental events occurring near pupation and pupariation in 32 species of *Drosophila* and two non-drosophilid species. The table lists the time of pupation and apocrine secretion, whether Sgs-granules and glue are present, the relative intensity of detection of BR-C and p127 proteins using D. melanogaster-specific antibodies, the species‘ preferred site of pupariation, the type of food used in this study, the number of secretory cells per SG lobe, whether collector sacculae, post-apocrine concrements, and long anterior spiracles are present, and the geographical occurrence (originally reported or currently known) of a particular species. The times of pupation and apocrine secretion for a species are the range of times seen in three sets of observations, each having a minimum of 30 animals. The number of secretory cells for a species is the arithmetic means of cell counts obtained using a fluorescent microscope and a 20× objective lens for a minimum of 15 pairs of SGs from each species, in triplicates. Abbreviations: APF = after puparium formation; Legend for symbols: Relative intensity of fluorescence is depicted by the number of plus (+) signs for each of the proteins, where five pluses (+++++) represents the strongest signal, a signal equal to that found in D. melanogaster, and a single plus (+) indicates the weakest signal. In additional columns a single plus (+) indicates the presence of collector sacculae, the presence of concrements, and the formation of long spiracles during pupariation, respectively. An empty cell in these columns indicates the absence of collector sacculae, concrements, or long spiracles. Negative signs (−) in these columns were omitted for clarity.

	Species	Food type	Pupariation site	Sgs glue	Time of pupation^a^ ^,^ ^b^	BR-C/p127 staining^c^	Apocrine secretion^a^ ^,^ ^b^	Cells/gland^d^	Sacculae^e^	Concrement^e^	Long spiracles^e^	Geographical occurrence
1	*D*. *melanogaster*	Molasses	Glass wall	Yes	13 h APF	+++++/++++	8–10 h APF	134				Africa, cosmopolitan
2	*D*. *simulans*	Molasses	Glass wall	Yes	11–12 h APF	+++/++	8–10 h APF	121				Africa, cosmopolitan
3	*D*. *montana*	Sugar	Glass wall/paper	Yes	17 h APF	++/++++	11–13 h APF	89				North America
4	*C*. *costata*	Sugar/malt	Glass wall/paper	No	21–22 h APF	++/++++	18–19 h APF	48				Japan
5	*D*. *hydei*	Molasses	Glass wall	Yes	17 h APF	+/++	11–13 h APF	110			+	cosmopolitan
6	*D*. *affinis*	Ban-opuntia	In food	No	11 h APF	+/++	8–10 h APF	111				North America
7	*D*. *bipectinata*	Molasses	In food	Yes	11.5 h APF	++/++++	7–9 h APF	82				Southeast Asia
8	*D*. *willistoni*	Molasses	In food	No	10–12 h APF	++/++++	11–13 h APF	78		+		South America, Brazill
9	*D*. *ananassae*	Molasses	Glass wall	Yes	11–13 h APF	++/++	10–11 h APF	75				Southeast Asia
10	*D*. *pseudoananassae*	Molasses	In food	Yes	11 h APF	+++/++++	7–9 h APF	86				Southeast Asia
11	*D*. *yakuba*	Molasses	Glass wall	Yes	11 h APF	+++++/++++	8–9 h APF	109				West Africa; cosmopolit
12	*D*. *virilis*	Molasses	Glass wall/paper	Yes	14.5 h APF	++/++++	12–13 h APF	82				East Asia
13	*D*. *atripex*	Molasses	Food/glass wall	No	12 h APF	+++/++++	8–10 h APF	104				Malacca, Malaysia
14	*D*. *parabipectinata*	Molasses	Glass wall/food	Yes	10–12 h APF	++/++++	8–10 h APF	99				Southeast Asia
15	*D*. *pallidosa*	Molasses	Prefer in food	Yes	12–13 h APF	++/++++	9–11 h APF	92				Fiji, Oceania
16	*D*. *mauritiana*	Molasses	In food	Yes	12.7 h APF	+++/++++	8–10 h APF	107				Africa, Mauritius
17	*D*. *erecta*	Molasses	In food	Yes	13.4 h APF	+++++/++++	9–11 h APF	115	+	+		West Africa
18	*D*. *lebanonensis*	Ban-opuntia	Prefer in food	Yes	19.5 h APF	+++/+++	16–18 h APF	106				North America
19	*D*. *funebris*	Molasses	Glass wall	Yes	14.5 h APF	+++/++++	13–14 h APF	96			+	cosmopolitan
20	*D*. *mojavensis*	Molasses	Glass wall	Yes	11 h APF	+++/++	7–9 h APF	105		+		Mojava Desert, CA, United States
21	*D*. *sechellia*	Molasses	Food/glass wall	Yes	13.2 h APF	+++++/++++	11–12 h APF	108				Africa, Seychelles
22	*D*. *pseudoobscura*	Molasses	Food/glass wall	Yes	18–19 h APF	++/+++	15–17 h APF	69				Northeast United States
23	*D*. *persimilis*	Ban-opuntia	In food	Yes	19 h APF	++/+++	15–16.5 h APF	110				Eastern North America
24	*D*. *albomicans*	Molasses	In food	No	12 h APF	+++/++++	10–11 h APF	106	+	+	+	China and Southeast Asia
25	*D*. *eugracilis*	Molasses	In food	Yes	14 h APF	++/++	11–12 h APF	133		+		India and Southeast Asia
26	*D*. *subobscura*	Molasses	In food	Yes	16 h APF	+++/++	13–15 h APF	84		+		Palearctic; cosmopolitan
27	*D*. *birchii*	Molasses	Food/glass wall	Yes	15 h APF	+++/++++	12.5–13.5 h APF	114	+			New Guinea, Australia
28	*D*. *sulfurigaster*	Molasses	Prefer in food	Yes	12 h APF	++/++	11 h APF	116	+	+	+	China and Southeast Asia
29	*D*. *equinoxialis*	Molasses	Glass wall	Yes	13.5 h APF	+++/++	12–12.5 h APF	84	+	+		Central and South America
30	*D*. *immigrans*	Molasses	Glass wall	No	14 h APF	+++/++	13 h APF	87	+	+	+	East Asia, Australia, Latin America; cosmopolitan
31	*D*. *busckii*	Ban-opuntia	Prefer in food	No	12 h APF	++/++++	10–11 h APF	70	+			Burma and India
32	*D*. *robusta*	Ban-opuntia	Food/glass wall	No	18–18.5 h APF	++/+++	15–17 h APF	117			+	Japan; cosmopolitan
33	*Lucilia cuprina*	Beef liver	In soil	No	12–16 h APF	++/++++	18.5–22.5 h APF	426				Australia
34	*Hermetia illucens*	Catering waste	Away from food	No	11–12 days APF	++/++++	10–11 days APF	164				cosmopolitan

^a^ The table lists the time of pupation and apocrine secretion, whether Sgs-granules and glue are present, the relative intensity of detection of BR-C and p127 proteins using *D*. *melanogaster*-specific antibodies, the species-preferred site of pupariation, the type of food used in this study, the number of secretory cells per SG lobe, whether collector sacculae, post-apocrine concrements, and long anterior spiracles are present, and the geographical occurrence (originally reported or currently known) of a particular species.

^b^ The times of pupation and apocrine secretion for a species are the range of times seen in three sets of observations, each having a minimum of 30 animals.

^c^ The relative intensity of fluorescence is depicted by the number of plus (+) signs for each of the proteins, where five pluses (+++++) represents the strongest signal, a signal equal to that found in *D*. *melanogaster*, and a single plus (+) indicates the weakest signal.

^d^ The number of secretory cells for a species is the arithmetic means of cell counts obtained using a fluorescent microscope and a 20× objective lens for a minimum of 15 pairs of SGs from each species, in triplicate.

^e^ A single plus (+) indicates the presence of collector sacculae, the presence of concrements, or the formation of long spiracles during pupariation. An empty cell in these columns indicates the absence of collector sacculae, concrements, or long spiracles. Negative signs (-) were not used for clarity.

To extend our knowledge about the timing of apocrine secretion in these species, we initially screened prepupal SGs from a species by antibody staining in groups of animals that were between 5 and 2 h from pupation. If no signs of secretion were found, we extended the time scale 1 h in both directions, and repeated the screening process. We continued this until we found signs of a positive secretion in the lumen of the SGs. At that point, the time scale was extended by 1 h in both directions, and the entire protocol was repeated once again to determine the exact duration of the entire apocrine secretory process.

In addition to these parameters, we also examined whether a wandering phase occurred, the preferred location for pupariation (food vs. vial wall), and the ability of the animal to become cemented *via* a Sgs to the vial wall or a folded filter paper.

### Orthology-based selection of antibodies appropriate to detect apocrine secretion

**FIGURE 1:**
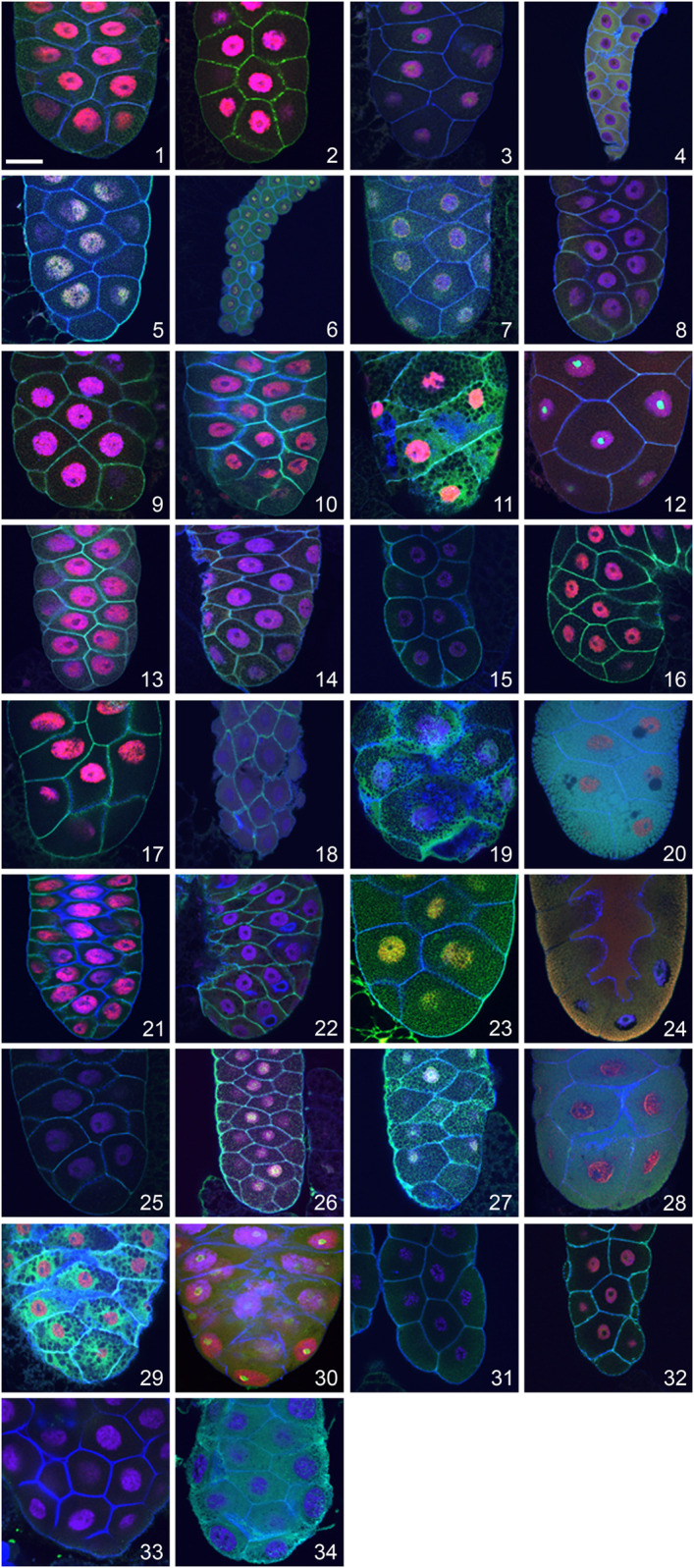
Visualization of late larval SGs of 32 *Drosophila* species and two unrelated dipteran species showing the presence and distribution of the transcription factor Broad-Complex (BR-C; red), the cytosolic cytoskeletal tumour suppressor protein p127^l(2)gl^ (green), filamentous actin (blue) and nuclear DNA (violet). In many species, the distribution of the p127^l(2)gl^ protein and actin allows the detection of Sgs-granules as numerous “black dots” in the SG cytoplasm. This is most prominent in *Drosophila yakuba* (11) and *Drosophila equinoxialis* (29). A unique situation was observed in *Drosophila mojavensis* (20), *Drosophila sulfurigaster* (28), and *Drosophila albomicans* (24) where p127^l(2)gl^ protein becomes part of secretory granules, highlighting them as numerous individual green and/or yellowish-green vesicles filling the entire cytoplasm. In several species with minimal cytoplasm and large nuclei, no Sgs-granules were detected (*Drosophila pallidosa* (15), *Chymomyza costata* (4), *Drosophila lebanonensis* (18), or *Drosophila busckii* (31). As presented in Table 1, some species show lower cross-reactivity to the antibodies we used, which resulted in a weaker fluorescent signal. The complete set of species shown here is: (1) *Drosophila melanogaster*, (2) *Drosophila simulans*, (3) *Drosophila montana*, (4) *Chymomyza costata*, (5) *Drosophila hydei*, (6) *Drosophila affinis*, (7) *Drosophila bipectinata*, (8) *Drosophila willistoni*, (9) *Drosophila ananassae*, (10) *Drosophila pseudoananassae*, (11) *Drosophila yakuba*, (12) *Drosophila virilis*, (13) *Drosophila atripex*, (14) *Drosophila parabipectinata*, (15) *Drosophila pallidosa*, (16) *Drosophila mauritiana*, (17) *Drosophila erecta*, (18) *Drosophila lebanonensis*, (19) *Drosophila funebris*, (20) *Drosophila mojavensis*, (21) *Drosophila sechellia*, (22) *Drosophila pseudoobscura*, (23) *Drosophila persimilis*, (24) *Drosophila albomicans*, (25) *Drosophila eugracilis*, (26) *Drosophila subobscura*, (27) *Drosophila birchii*, (28) *Drosophila sulfurigaster*, (29) *Drosophila equinoxialis*, (30) *Drosophila immigrans*, (31) *Drosophila busckii*, (32) *Drosophila robusta*, (33) *Lucilia cuprina*, and (34) *Hermetia illucens*. In all of the confocal images, the posterior end of the gland is oriented down. The scale bar in the lower left corner represents 50 microns.

To screen for the presence of apocrine secretion within the *Drosophilidae* family, we used a panel of antibodies some of which were used also in our initial description of the apocrine process in *D*. *melanogaster* (Farkaš et al., 2014), able to detect orthologous proteins in species having varying evolutionary distances from *D*. *melanogaster*. To select antibodies from those in our collection we made detailed BLAST and FASTA comparisons of specific proteins among the *Drosophila* species covered by genome sequencing projects. BLAST and FASTA analyses of protein sequences were run with the Wisconsin GCG package 10.1 installed on Sun Fire 280 R under Solaris 5.8.2 or Husar/GCG software run on Sparc SUNW Sun-Fire-880 R under Solaris 5.9. The FlyBase BLAST of *D*. *melanogaster* sequences also was run against *v*. FB 2017-05, release 1.04 fly sequence databases (dos Santos et al., 2015; Hoskins et al., 2015; Thurmond et al., 2019). Based on these comparisons, we selected antibodies for the l(2)gl^p127^ and BR-C proteins to use in screening, as these proteins have shown a particularly high degree of interspecies conservation of the peptide sequences that were used to generate antibodies, so provide a higher likelihood that antibodies generated against *D*. *melanogaster* epitopes will be able to detect orthologues in other *Drosophila* species, including those species whose genome has not been sequenced. Antibodies for these two proteins also have long-term availability. Before screening for apocrine secretion was started, we verified that these antibodies showed an expected pattern of staining in the larval salivary glands, fat bodies, CNS, foreguts/midguts and imaginal discs of all species under study. To document these results, we show staining of the SGs (the tissue under study) here (Figure 1). These experiments also allowed us to discern whether the formation of glue Sgs-granules occurs in the larval SGs of a particular species.

### Immunohistochemistry and confocal microscopy

SGs were dissected under stereomicroscope in *Drosophila* saline solution and fixed in Pipes-buffered 4% paraformaldehyde (20 mM Pipes, 60 mM sucrose, 1 mM EGTA, 5 mM MgCl_2_, pH 7.2). Since *L. cuprina* has extremely fast coagulation and melanization, these unwanted processes were inhibited during dissection by supplementing saline with saturated phenylthiourea and 1 mM EDTA. Prior to staining tissues with antibodies, they were permeabilized with 0.1% Triton X-100 in PBS (PT) and then blocked with PT containing 2% fraction V of bovine serum albumin (Serva) (PBT) and 2% goat serum (Sigma). After blocking tissues were incubated overnight at 4°C with primary antibodies: rabbit anti-p127 as well as mouse anti-BR-C. To detect primary antibodies, Cy3-conjugated anti-rabbit and Cy5-conjugated anti-mouse affinity purified F(ab’)_2_ specific pre-absorbed secondary antibodies were used (Jackson ImmunoResearch Laboratories, Inc.) diluted 1:200. Actin was detected using AlexaFluor_488_-or AlexaFluor_546_-phalloidin (Molecular Probes Inc.) at 0.04 nM concentration. Depending on the fluorochrome combination for antibodies and phalloidin, nuclei were counterstained for DNA either with 5 μg/ml Hoechst-33258 (Calbiochem), 0.5 μg/ml Oli-Green or 0.1 μg/ml Toto-3 (both Molecular Probes Inc.). After extensive destaining in PT solution, tissues were mounted in Dabco-supplemented Elvanol and scanned on a Zeiss LSM-510 Meta laser confocal microscope using 40 × (oil NA 1.3) lenses. The intracellular or lumenal distribution of representative proteins was quantified by measuring the fluorescence signal [Cy5 (633 nm); Cy3 (546 nm); AlexaFluor_488_ (488 nm)]. Fluorescence intensity was evaluated by using Histogram module of Zeiss AIM LSM5 application as described previously (Farkaš et al., 2014). The bitmap images obtained were processed in Adobe PhotoShop and assembled into figures in Aldus FreeHand or Corel Draw software. Altogether more than 11,000 salivary glands were examined, and more than 7,500 confocal images were obtained.

In all species, the numbers of secretory cells (Hoechst-positive polytene nuclei) were counted under fluorescence microscope (Leica DMR-E) equipped with an A-type prism cube using a 20× objective lens. A minimum 15 pairs of SGs in triplicate were counted for each species. The average values are presented in Table 1.

Wide-field light microscope observations of the SGs were made and images were taken on Leica DM6000B microscope with a Leica DFC480 color camera controlled *via* LAS 2.6.1 software.

## Results

### Visualizing apocrine secretion across the *Drosophilidae*

Multi-channel confocal microscopy was used to follow representative cytoskeletal (filamentous actin), cytosolic (tumor suppressor p127^l(2)gl^) and nuclear (BR-C) protein markers to visualize the fate of cytoskeletal, cytosolic and nuclear cellular components during apocrine secretion. Filamentous actin was readily detected across species using fluorescently conjugated phalloidin. The fate of the cytosolic fraction was followed by using a rabbit anti-p127 polyclonal antibody, which recognizes the tumor suppressor protein produced by *lethal (2) giant larvae* (*l(2)gl*), a gene having orthologues in all so-far sequenced *Drosophila* and other insect species (Jacob et al., 1987; Strand et al., 1994), as well as mammals (Strand et al., 1995). The nuclear fraction was followed using a mouse monoclonal antibody against the abundant ecdysone-regulated transcription factor BR-C, encoded by a *Broad-Complex* locus (DiBello et al., 1991; von Kalm et al., 1994), which also has orthologues in all so-far sequenced *Drosophila* and other insect species. Before screening the prepupal glands of non-*melanogaster* species for apocrine secretion, we tested whether the two antibodies stained their larval SGs in the expected pattern. If a SG signal was absent or very poor, we evaluated staining in additional larval organs such as the fat body, CNS, foregut/midgut and imaginal discs. Larval organs were chosen because they are more easily dissected than those from prepupal stages, previous studies in *D*. *melanogaster* have documented that the targets of these antibodies are abundantly present in both the late 3rd larval instar and the prepupal organs (Emery et al., 1994; Strand et al., 1994; Mugat et al., 2000), and they were successfully used in our previous investigations of apocrine secretion (Farkaš et al., 2014; Beňová-Liszeková et al., 2021). Table 1 summarizes comparative data obtained from 34 species. The relative intensity of fluorescence is indicated by the number of plus (+) signs: five (+++++) signifies the strongest signal, equal to that found in *D*. *melanogaster*, one (+) the weakest signal, and a minus (−) indicates that no signal was present. Both proteins were easily detected in the larval SGs of more than half of the species at (++) or (+++) levels, suggesting that they would be detectable also in the species’ prepupal stages. Indeed, while the p127 protein was unambiguously detected in the larval SGs of all species studied, the BR-C protein was detected in the larval SGs of all species except for *C*. *costata* and *L*. *cuprina*. In *C*. *costata,* BR-C was detected in larval organs only in the optic lobes and the eye imaginal discs. In *L*. *cuprina* BR-C was not detected in any larval organ. As will be described below, however, BR-C was detected in the late prepupal SGs of both species. In all species, the combination of visualizing cortical filamentous actin and the cytosolic p127 protein provided an excellent means to visualize the presence of Sgs-granules in the larval SG, as these appear as “black dots” within a dense reticulate meshwork (Figure 1). When the Sgs-granules were absent in a particular species, only a diffuse signal was seen over the entire cytoplasm, and no “black dots” were observed.

### The exocytotic release of salivary gland glue is not evolutionarily conserved in the *Drosophilidae*

We systematically characterized the complex set of changes associated with metamorphosis in *Drosophila* species that have a direct relationship to the function of their SGs by quantifying the preferred site of pupariation, the occurrence of Sgs-granules, the number of SG cells per lobe, and the length of the anterior spiracles. We selected these features since they seemed likely to be strongly associated with SG function, specifically with the production and volume of the apocrine secretion as well as that of the Sgs-glue released by exocytosis. Furthermore, we also selected these features since characterizing them would allow us to address three unanswered questions: Is apocrine secretion a novel property of the SGs of *D*. *melanogaster* and perhaps closely related species? Does Sgs-glue production occur in all *Drosophila* species? Since in *D*. *melanogaster* apocrine secretion follows the secretion of the Sgs glue, is apocrine secretion somehow coupled to the production and exocytosis of Sgs-glue? Characterizing features such as the site of pupariation or size/extension of everted anterior spiracles would provide insight into these questions, since they are all linked to one developmental event. Although the number of future secretory SG cells is established during mid-embryogenesis, the cementing function of the Sgs-glue produced by these cells as well as the extension of the anterior spiracles takes place at the moment of pupariation.

As summarized in Table 1, we found that nine species either did not produce any Sgs-granules or showed very diminished production of such granules (*C*. *costata*, *D*. *affinis*, *D*. *willistoni*, *D*. *atripex*, *D*. *mauritiana*, *D*. *albomicans*, *D*. *immigrans*, *D*. *busckii*, and *D*. *robusta*). Interestingly, larvae of 12 species failed to leave the food and crawl up the side of the culture vial, so they pupariate within the culture medium itself (*D*. *bipectinata*, *D*. *willistoni*, *D*. *pseudoananassae*, *D*. *pallidosa*, *D*. *erecta*, *D*. *lebanonensis*, *D*. *persimilis*, *D*. *albomicans*, *D*. *eugracilis*, *D*. *subobscura*, *D*. *busckii*, *D*. *parabipectinata*). Six species formed quite long anterior spiracles during pupariation that were not visible in their larvae: *D*. *hydei*, *D*. *funebris*, *D*. *albomicans*, *D*. *sulfurigaster*, *D*. *immigrans*, and *D*. *robusta*. Among these, three species did not have any Sgs-granules or glue secretion (*D*. *albomicans*, *D*. *immigrans*, and *D*. *robusta*). Only one of these three species pupariated within the food (*D*. *albomicans*). Therefore, an absence of Sgs-granules *per se* is not directly linked to a species’ preference to pupariate in the food, and the length of anterior spiracles which could be used to facilitate breathing while embedded in such an environment is not directly or exclusively linked to a mode of pupariation within food. In contrast, some of the species that pupariated in the food produced evident Sgs-granules and released Sgs-glue (*D*. *bipectinata*, *D*. *pseudoananassae*, *D*. *mauritiana*, *D*. *pallidosa*, *D*. *erecta*, *D*. *sechellia*, *D*. *lebanonensis*, *D*. *persimilis*, *D*. *eugracilis*, *D*. *subobscura*, *D*. *busckii*, *D*. *birchii*). This raises the possibility that the Sgs-secretion may have another function in addition to serving as a glue, and that its service as a glue is a novel evolutionary specialization.

Nevertheless, at least three species of *Drosophilidae* did not have or use Sgs-glue to affix themselves to a substrate. In *C*. *costata*, *D*. *montana*, and *D*. *virilis*, larvae chewed up and ruminated filter paper using their mouth armature to liberate cellulose fibers that subsequently became attached in an irregular manner to the surface of the puparial cuticle. We observed that a varying number of larvae of two other species (*D*. *funebris* and *D*. *robusta*) facultatively displayed a similar behavior. In *C. costata* we observed that most mature 3^rd^ instar larvae did not undergo the typical wandering stage seen in other species: these animals preferred to climb out of the food only during the 2 h period of darkness after which they quickly pupariated and initiated rapid tanning of their puparium.

Further support of the idea that the Sgs secretion has evolved to allow for the affixing the puparia to a substrate is finding that the number of polytenized secretory cells per gland lobe and the production of Sgs-glue granules are positively correlated. Species with fewer polytenized secretory cells (65–90 per gland-lobe) such as *D*. *bipectinata*, *D*. *ananassae*, *D*. *willistoni*, *D*. *virilis*, and *D*. *pseudoobscura*, and in particular *C*. *costata*, which has only 48 cells per gland-lobe do not produce any Sgs-granules or glue. In contrast, species that produce a visibly large amount of Sgs-glue, such as *D*. *melanogaster*, *D*. *simulans*, *D*. *sechellia*, *D*. *mauritiana*, *D*. *yakuba*, *D*. *erecta*, *D*. *eugracilis*, have a higher number of cells (100–134) per gland lobe.

### Variation in the timing of pupation in the family *Drosophilidae*

Based on our original discovery that apocrine secretion in the SGs of *D*. *melanogaster* takes place 3–5 h prior to pupation (Farkaš et al., 2014), we hypothesized that any analogous secretory process, if present, should occur in a parallel timeframe, and that apocrine secretion and pupation will occur in close temporal proximity in non-*melanogaster* species. This was strongly suggested by our most recent finding that in addition to providing a clear immune-related and microbial-defense function, the apocrine secretion itself also serves as the exuvial fluid (Beňová-Liszeková et al., 2021). Therefore, we determined the time of pupation relative to puparium formation for each species we studied. To do so, we carefully selected animals at the white puparium (0 h prepupa, 0 h APF) stage. Newly formed puparia, after a brief rinsing, were placed onto a clean glass microscope slide inside a humid chamber, and closely examined using a Wild/Leica MZ-9^.5^ stereomicroscope. Animals were evaluated hourly during the first 6 h, and then every 15 min thereafter until the sharp contours of the everted pupal head and expelled larval mouth hooks could be observed, as these features demarcate the beginning of the pupal stage. The time when this occurred was recorded as the time of pupation. A minimum of 30 prepupae, in triplicate, were used to determine the exact time of pupation for each species under study.

Although every species has a unique time of pupation, two major groups are recognizable (Table 1). In the first group lie most species belonging to the *melanogaster* species group (including the *ananassae* subgroup or *saltans-willistoni* clade). Regardless of their body size or the number of secretory cells per SG lobe, they all have relatively similar times of pupation, from 11 to 14 h APF. Interestingly, this group includes some larger species originating in Oceania or Southeast Asia. In contrast, pupation is delayed in a second group where it occurs from 15 to 22 h APF. This group includes several Nearctic and Palearctic species (*D*. *lebanonensis*, *D*. *pseudoobscura*, and *D*. *robusta*, the *virilis-repleta* radiation, which migrated to the New World from Japan, *D*. *montana* and *C*. *costata*). The majority, albeit not all of these species are larger than *D*. *melanogaster*. There is, however, no clear-cut correlation in this group between the time of pupation, body size, or the number of secretory cells per SG lobe.

### Apocrine secretion occurs in both *Drosophila* and non-*Drosophila* species

After determining when pupation occurred in each species, we screened each species for the occurrence of apocrine secretion. To localize when apocrine secretion occurs, we screened animals at times between five and 2 h prior to pupation. If no signs of secretion were present, we iteratively extended screening times by 1 h in both directions. After signs of positive apocrine secretion were found in the SG lumen, we defined the interval over which the entire secretory process occurred by iteratively extending screening times by an additional hr in both directions (Table 1).

**FIGURE 2:**
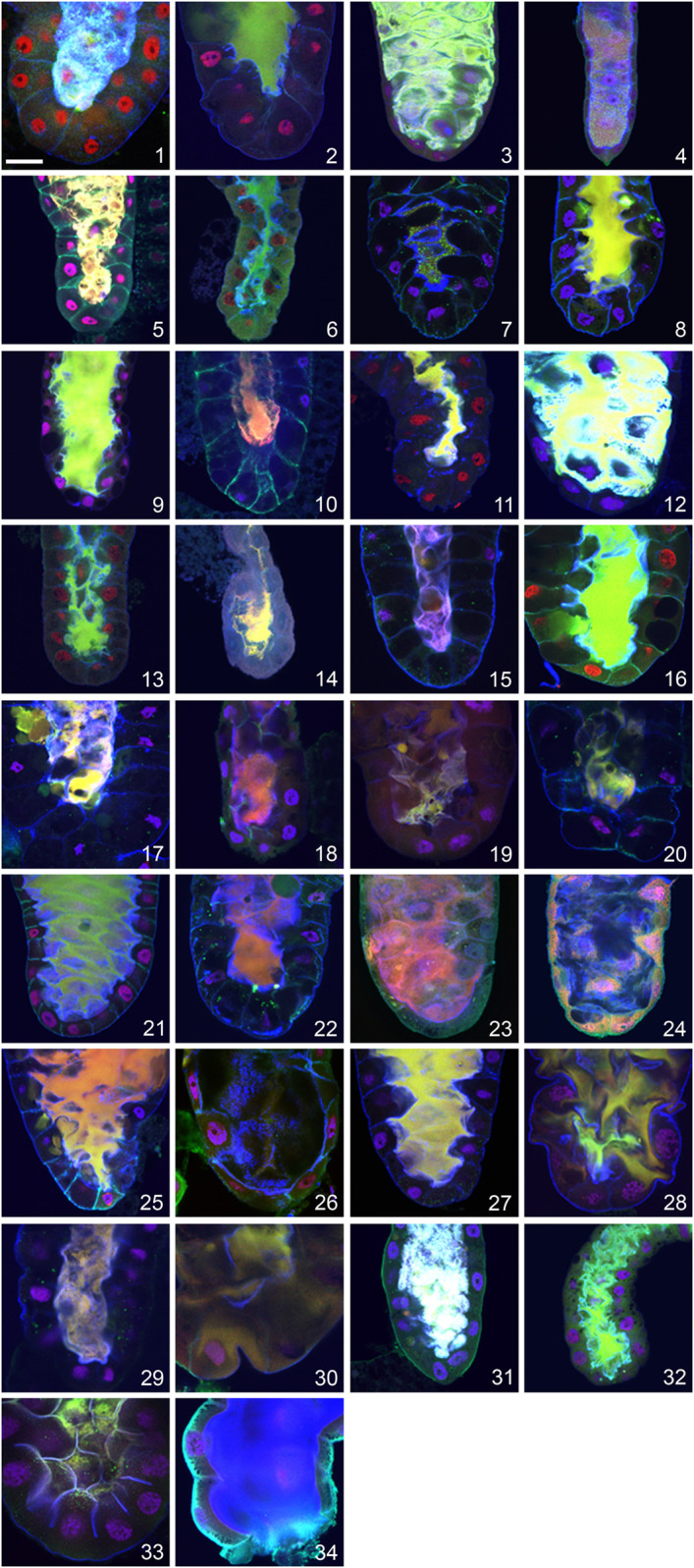
Evidence for apocrine secretion in the prepupal SGs of 32 species of family *Drosophilidae* and two unrelated dipterans documented as a positive fluorescent signal released into centrally located lumen. (1) *D. melanogaster*, (2) *D. simulans*, (3) *D. montana*, (4) *C. costata*, (5) *D. hydei*, (6) *D. affinis*, (7) *D. bipectinata*, (8) *D. willistoni*, (9) *D. ananassae*, (10) *D. pseudoananassae*, (11) *D. yakuba*, (12) *D. virilis*, (13) *D. atripex*, (14) *D. parabipectinata*, (15) *D. pallidosa*, (16) *D. mauritiana*, (17) *D. erecta*, (18) *D. lebanonensis*, (19) *D. funebris*, (20) *D. mojavensis*, (21) *D. sechellia*, (22) *D. pseudoobscura*, (23) *D. persimilis*, (24) *D. albomicans*, (25) *D. eugracilis*, (26) *D. subobscura*, (27) *D. birchii*, (28) *D. sulfurigaster*, (29) *D. equinoxialis*, (30) *D. immigrans*, (31) *D. busckii*, (32) *D. robusta*, (33) *L. cuprina*, and (34) *H. illucens*. The following proteins were detected in all species: red = transcription factor Broad-Complex, green = cytosolic and cytoskeletal tumour suppressor protein p127^l(2)gl^, blue = filamentous actin. Nuclear DNA is depicted in violet. In all confocal images the posterior end of the SG oriented down. The scale bar in the lower left corner represents 50 microns.

The most exciting result of these analyses was that, in contrast to production of Sgs-glue and its exocytosis, apocrine secretion was unambiguously present in all 34 species we studied, regardless of their climatic or geographic region, habitat and phylogenetic relatedness (see Figure 2). Moreover, we observed apocrine secretion in two unrelated dipteran species, *L. cuprina* (family *Calliphoridae*) and *H. illucens* (family *Stratiomyidae*), which suggests that the utilization of apocrine secretion from the prepupal SGs to produce exuvial fluid is likely to be evolutionarily conserved throughout the order *Diptera*.

Nevertheless, we did observe species-specific characteristics in the appearance and/or intensity of apocrine secretion. If *D*. *melanogaster* is considered as an etalon species, some species show a more massive secretion with a stronger luminal signal, while others show a more minor secretion and a weaker luminal signal. The first group, which includes *D*. *melanogaster*, *D*. *simulans*, *D*. *sechellia*, *D*. *ananassae*, *D*. *virilis*, and *D*. *montana*, is characterized by a strong signal in the entire, and often wider, lumen for a period of at least 60 min, whereas the second group, which includes *D*. *yakuba*, *D*. *pallidosa*, *D*. *subobscura*, *D*. *funebris*, *D*. *parabipectinata*, *D*. *lebanonensis*, *D*. *mojavensis*, and *C*. *costata*, is characterized by a less abundant fluorescence signal that is mostly restricted to the posterior, middle or anterior portion of a narrow lumen, and is there for only a relatively short period (15–20 min). It was very unusual to find a SG lumen completely filed with secretion in these species. We did not consider differences in fluorescence intensity, which could reflect the amount of the assayed protein released into the SG lumen by apocrine secretion, to be an important feature to quantify for our purposes here, as these could reflect species-specific differences in the recognition of the target antigen by the primary antibody as well as variation in the SG expression of that protein. This should be addressed in an independent study.

In most species, apocrine secretion occurs 2 to 3 h prior to pupation, as it does in *D*. *melanogaster*. However, there are notable exceptions to this general finding. For example, in *D*. *montana* and *D*. *hydei* it occurs between four and 6 h prior to pupation. In contrast, there are species where apocrine secretion occurs within 1 h prior to pupation (*D*. *funebris*, *D*. *ananassae*, *D*. *pallidosa*, *D*. *subobscura*, *D*. *sulfurigaster*, *D*. *equinoxialis*, *D*. *albomicans*, *D*. *eugracilis*, *D*. *robusta*). Strikingly, it does not always occur prior to pupation, as it occurs in *D*. *parabipectinata* during pupation and occurs postpupation in *L. cuprina*.

**FIGURE 3:**
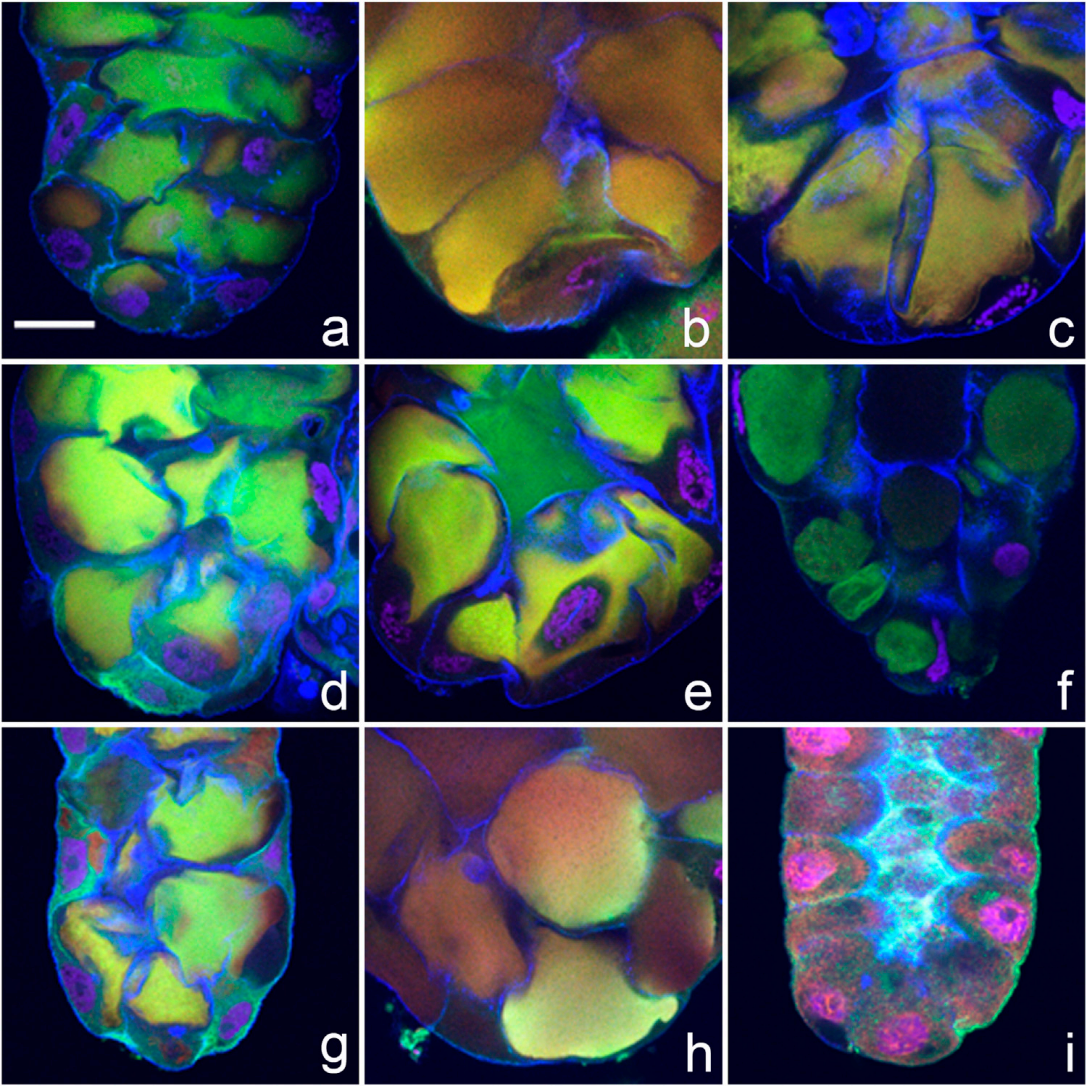
Pre-apocrine collector sacculae are present in the prepupal SGs of eight species of *Drosophila*. **(A)** *D. sulfurigaster*, **(B)** *D. immigrans*, **(C)** *D. albomicans*, **(D)** *D. eugracilis*, **(E)** *D. birchii*, **(F)** *D. busckii*, **(G)** *D. mojavensis*, and **(H)** *D. virilis*. In *D. albomicans*, the final phases of sacculae contents release are slower, and some signal associated with secreted proteins can be still detected in the lumen, even after the gland itself has attained its previous smaller size **(I)**. The following proteins were detected in all species: red = transcription factor Broad-Complex, green = cytosolic and cytoskeletal tumour suppressor protein p127^l(2)gl^, blue = filamentous actin. Nuclear DNA is depicted in violet. In all confocal images the posterior end of the gland is oriented down. The scale bar in the lower left corner of **(A)** represents 50 microns.

We observed a new and distinctive feature of apocrine secretion in a small number of species: the presence of pre-apocrine sacculae (“collector” organelles) where future secretory material is concentrated for a period of about 2 h prior to when it is abruptly released into the SG lumen. This phenomenon occurred in *D*. *erecta*, *D*. *albomicans*, *D*. *birchii*, *D*. *sulfurigaster*, *D*. *equinoxialis*, *D*. *immigrans*, *D. virilis*, and *D*. *busckii* (see Figures 3A–H). These sacculae were most prominent in *D*. *albomicans* and *D*. *sulfurigaster*, and at times one or two large sacculae appeared to entirely fill a cell’s volume. In multi-channel confocal microscopy visualizing cytoskeletal, cytosolic, and nuclear protein markers, these sacculae were identifiable by a light-green, yellow, or even white color that reflects the merged signal of three colocalized markers. Except for some residual cortical actin, the marker proteins were not detected in their original cellular locations. When this form of presecretory storage was present, the prepupal SGs were wider. In most species where sacculae developed, the subsequent process of secretion into the SG lumen was very fast—it appeared to be completed within 5–10 min. An exception was found in *D*. *albomicans*, where the final phase of sacculae contents release was slower, and signal associated with the secreted proteins was still detected in the lumen even after the gland had returned to its earlier smaller size (see Figure 3I).

**FIGURE 4:**
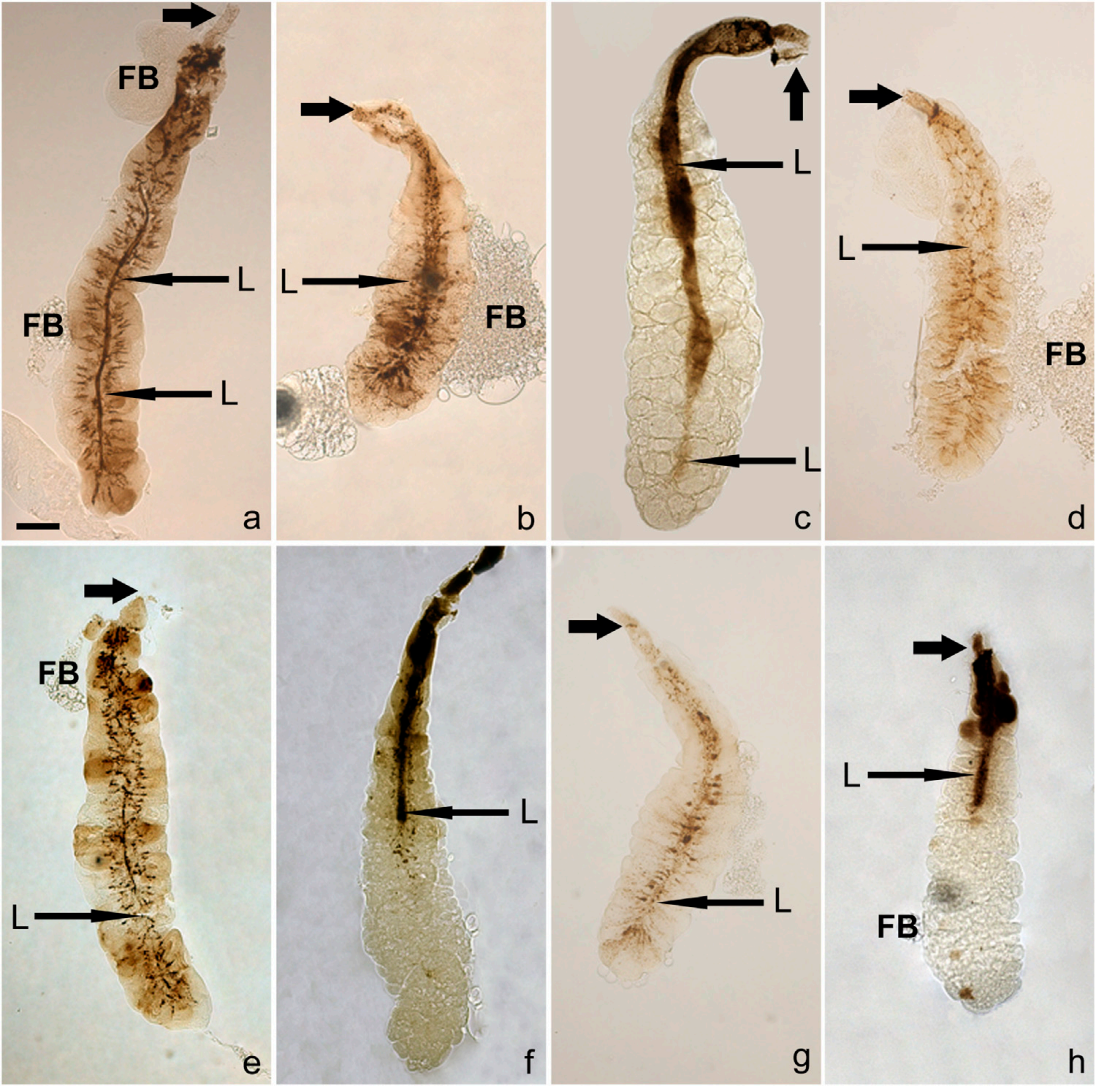
Post-apocrine concrements are found in the lumens of very late prepupal and/or early pupal SGs of eight species **(A)** *D. willistoni*, **(B)** *D. subobscura*, **(C)** *D. mojavensis*, **(D)** *D. albomicans*, **(E)** *D. eugracilis*, **(F)** *D. sulfurigaster*, **(G)** *D. immigrans* and **(H)** *D. equinoxialis*. Long arrows indicate centrally located lumen (L), whereas short thick arrows point to anterior ducts. The scale bar in the lower left corner of **(A)** represents 100 microns. Legend: D, duct, FB, adhering fat body.

**FIGURE 5:**
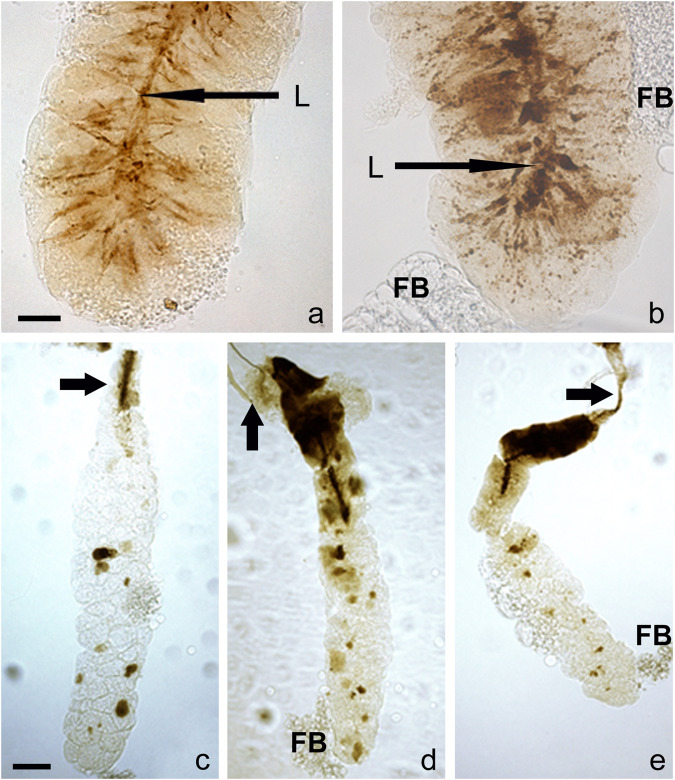
A detailed view of the concrements is some species reveal that they form stream-like structures within the cell cytoplasm connecting to a centrally located belt (lumen). These resemble a dense network akin to a river-delta in **(A)** *D. willistoni* and **(B)** *D. subobscura*. As documented in these images, this dark and opaque material is released in a posterior-to-anterior direction; when no additional opaque material is produced by the cell, the lumen becomes cleared from its posterior end. In *D. albomicans* **(C)**, *D. mojavensis* **(D)**, and *D. equinoxialis* **(E)**, some dark brown material remains within the cytoplasm after most of this opaque material is released and delivered into the exuvial fluid. The scale bars in the lower left corner of **(A,C)** represents 25 and 100 microns, respectively. Legend: L, lumen, D, duct, FB, adhering fat body.

An additional and unexpected attribute of very late prepupal or early pupal SGs was seen when bright-field images of unstained SGs were examined: in their lumen were brownish to dark brown post-apocrine concrements. These resembled a dense river-delta network, appearing as stream-like structures within the cell cytoplasm that connect to a centrally located belt (lumen) (Figures 4, 5). These concrements were not seen in all species but were found in *D*. *willistoni*, *D*. *mojavensis*, *D*. *subobscura*, *D*. *albomicans*, *D*. *eugracilis*, *D*. *sulfurigaster*, *D*. *equinoxialis*, *D*. *immigrans* and irregularly also in *D*. *erecta* (Figures 4A–H). They became visible 1 h after the completion of apocrine secretion, so were not derived from material released during or *via* apocrine secretion. As illustrated in some of the SG images shown in Figures 4, 5, however, this material was also released in a posterior-to-anterior direction (Figures 4C, F, H, 5D, E). After no additional material was produced, the lumen was cleared anteriorly. Dark brown inclusions remaining inside the cytoplasm after most of this concremental material was released and transported into the exuvial fluid are apparent in Figures 4H, 5C–E.

Some species (*D*. *sulfurigaster*, *D*. *equinoxialis*, *D*. *immigrans*, *D*. *albomicans*, and *D*. *eugracilis*) share both the pre-apocrine sacculae (collector organelles) and the post-apocrine concrements. This suggests that there may be a causal relationship between the newly identified collector organelles and the post-apocrine concrements.

## Discussion

Apocrine secretion, substantially distinct from exocytosis, is poorly characterized transport and secretory mechanism that appears to be evolutionarily conserved among all metazoans including humans. After discovering this secretory mechanism in the *Drosophila* salivary glands (Farkaš et al., 2014), we use this model organism to genetically dissect and molecularly characterize this non-canonical and non-vesicular externalization pathway. We undertook this pivotal study to clarify the evolutionary relationship of apocrine secretion and the exocytotic release of Sgs-glue in the *Drosophilidae*. The most important conclusion of this work is that apocrine secretion by the prepupal SGs serves as the major evolutionarily conserved mechanism underlying the release of anti-microbial exuvial fluid across all of the examined species of the *Drosophila* genus. This includes species collected from geographical regions and habitats across five continents, as well as two distant dipteran species such as *L. cuprina* and *H. illucens*. These results also clearly document that the massive exocytotic secretion of glue prior to pupariation, which is typical for the *D. melanogaster*, is not present and conserved in the SGs of all *Drosophila* species. This indicates that the primary evolutionary function of this organ in fruitflies is connected to providing the pupae an active defense against microbial/viral invasion *via* the production of pupal exuvial fluid, and that the production of the Sgs is a specialized adaptation of this gland in a subset of *Drosophila* species, which could have evolved more recently than apocrine delivery.

This is in contrast to the dominant view for more than half a century that in the majority of dipterans, the major if not the only function of fully mature SGs is the production of a large amount of mucinous secretory granules that, when released just prior to pupariation, serve as a glue to affix a freshly formed puparia to a substrate. Historically, this view was supported by parallel studies in *D*. *melanogaster* and *Phormia regina* (Fraenkel and Brookes, 1953). After the Sgs secretory granules are synthesized during the second half of the last larval instar, the subsequent wandering larval phase allows the animal to seek an optimal place for pupariation. When that site is found, the Sgs-secretion is first released into the gland lumen by exocytosis, and then expectorated throughout the mouth. This study demonstrates that Sgs secretion is not the only, and not the most conserved function of the mature SGs.

When we started this study, among the first non-*melanogaster* species we studied was *C. costata*. We observed that their mature 3rd instar larvae climb out of the food only during a 2 h period of darkness—without any previous wandering stage—when they also quickly pupariate and initiate rapid tanning of their puparium. They tended to strongly prefer a piece of folded filter paper for pupariation and tried to avoid the glass wall of the vial. This hinted that they may not have enough or any Sgs glue in their SGs and so may not be undergoing any prepuparial exocytosis. *C. costata* proved to be an extraordinarily useful model system to uncover the independence of late prepupal apocrine secretion from the previous larval exocytosis. Indeed, it was this observation that prompted us to investigate Sgs-glue formation and granule presence in the late 3rd instar larva SGs of a wide range of species.

Moreover, our research has uncovered two other important and unexpected findings. First, since apocrine secretion can occur in species where exocytosis of the Sgs glue does not occur, from a mechanistic point of view, apocrine secretion may not depend on prior well-coordinated exocytosis events. Developmental observations in SGs of *D*. *melanogaster* (Farkaš et al., 2014) show that the larval exocytosis of Sgs-glue and the prepupal apocrine secretion are separated by at least 16 h. Since *D*. *melanogaster* development is relatively quick, this is a fairly long period. Many other previously neglected cellular activities occur in the SGs during this time (Farkaš et al., 2015; Farkaš et al., 2016). Our observations in *C. costata* indicate that it would be valuable to dissect the interdependence of exocytosis and apocrine secretion on each other using the molecular-genetic tools available in *D*. *melanogaster*.

Second, there is species-specific adaptation of SG function. A complex network of relationships exists between the properties of the larval/prepupal SGs in different *Drosophila* species and that species‘ wandering/prepuparial behavior. Consider the relationship between the intensity of Sgs-glue production, the amount of apocrine secretion and the number of SG cells. A paradoxical observation is that species with more intense Sgs-glue production and exocytosis in larvae (*D*. *melanogaster*, *D*. *simulans*, *D*. *yakuba*, etc.) show a much more profound and massive apocrine secretion at their late prepupal stage than those species that have no or only very minor Sgs-glue exocytosis (e.g., *C*. *costata*, *D*. *affinis*, *D*. *willistoni*, *D*. *atripex*, *D*. *mauritiana*, *D*. *albomicans*, *D*. *immigrans*, *D*. *busckii*, and *D*. *robusta*). Astonishingly though, is a positive correlation between the numbers of polytene secretory cells per gland lobe and production of Sgs-glue granules (see Table 1). Species such as *D*. *bipectinata*, *D*. *ananassae*, *D*. *willistoni*, *D*. *virilis*, and *D*. *pseudoobscura* have both a lower number of cells (65–90) per lobe and produce minimal Sgs-granules or glue—*C*. *costata* lies at one extreme with only 48 cells per gland lobe and does not produce any Sgs granules or glue. In contrast, species that produce higher amounts of Sgs-granules and glue show higher number of cells (100–134) per gland lobe (e.g., *D*. *melanogaster*, *D*. *simulans*, *D*. *sechellia*, *D*. *mauritiana*, *D*. *yakuba*, *D*. *erecta*, *D*. *eugracilis* etc.).

### *Sgs*-gene number, SG-cell number and exocytotic secretory activity evolve in parallel in the *Drosophilidae*

It is valuable to consider our results with respect to the distribution of *Sgs*-genes among sequenced *Drosophila* species (Adams et al., 2000; Celniker and Rubin, 2003; Birney, 2007; Clark et al., 2007; Heger and Ponting, 2007; Huntley and Clark, 2007; Lin et al., 2007; Stark et al., 2007; Minuk et al., 2011; dos Santos et al., 2015; Hoskins et al., 2015; Thurmond et al., 2019) and while reflecting on the latest molecular evolutionary study of *Sgs*-genes (Da Lage et al., 2019). The *Sgs3* and *Sgs5bis* genes are present in all sequenced *Drosophila* species, while the *Sgs4* and *Sgs1* genes are present in only some species. The *Sgs4* gene is *melanogaster* and *yakuba*-*erecta* group-specific. The *D*. *willistoni*, *D*. *virilis*, and *D*. *pseudoobscura* species groups appear to have only *Sgs3* or *Sgs3* and *Sgs7*, and rarely also the *Sgs5bis* gene, respectively. Species with fewer *Sgs-*genes (*Sgs3*, *Sgs5*/*Sgs5bis* or *Sgs3*, and *Sgs7*) do not necessarily affix themselves to a substrate, while the presence of a greater number of *Sgs*-genes (such as *Sgs1*, *Sgs4*, *Sgs8* along with *Sgs3*, *Sgs5*/*Sgs5bis*, and *Sgs7*) seems to be associated with evolving the ability to use components of larval SG secretion also for gluing to a substrate. There also appears to be a relationship between the number of *Sgs-*genes and the number of secretory cells in each SG lobe. Species with a lower number of secretory cells per gland lobe also harbor only one to three *Sgs*-genes (*Sgs3*, *Sgs5*/*Sgs5bis* and/or *Sgs7*) in their genome, whereas species with high number of cells per gland lobe contain 5 to 8 *Sgs*-genes per genome (also *Sgs1*, *Sgs4*, *Sgs8*, and *Eig71Ee*).

Hence, the duplication of *Sgs*-genes during species evolution appears to be a positive determinant of increasing the number of cells in each SG lobe and the ability to use an *Sgs*-secretion to affix pupae to a substrate. If this is the case, the number of secretory cells per SG lobe in an individual species is either determined by or co-regulated with, in some an unknown manner, the number and diversity of *Sgs*-genes within a species’ genome. This relationship parallels the rapidity of the evolved exocytotic secretory activity of the SG among more recently evolved *Drosophila* species. It will be valuable to investigate the regulatory basis of this relationship further: how is the number of highly specialized cells in a specific organ influenced by changes in the number of single-gene family members that are highly associated with one of its major functions.

How glue secretion itself evolved and whether it is an ancestral trait in some species that was lost and regained, perhaps multiple times, is an intriguing question that our data do not fully address. An evolutionary comparison of different species that have been sequenced reveals that some phylogenetically older species have fewer *Sgs*-genes (*D*. *virilis*, *D*. *willistoni*, *D*. *pseudoobscura*, *D*. *bipectinata*, and *D*. *ananassae* have two to three) than some phylogenetically younger species (*D*. *melanogaster*, *D*. *simulans*, *D*. *sechelia*, and *D*. *mauritiana* have five to seven *Sgs*-genes). Also, *Sgs*-genes are able to evolve quickly as there are conspicuous signs of accelerated evolution in the repeat regions of glue-gene proteins and there is intraspecific variation in the number of these repeats (Da Lage et al., 2019). These patterns, and the distribution of glue granules in some of the species reported here, not all of which have been sequenced, suggest the hypothesis that a *Drosophila* progenitor either did not produce glue or produced very little of it. We know that exocytosis in general is evolutionarily older than apocrine secretion, because exocytosis is present already in unicellular eukaryotes such as yeast. In comparison, apocrine secretion appears to be “limited” to metazoans. However, in the *Diptera*, we suggest that the massive apocrine secretion in the prepupal/early pupal SGs is older than massive merocrine secretion of the Sgs-glue. While exocytosis *per se* would have been present in dipteran SGs since their phylogenetical first appearance, the adoption of an exocytotic mechanism for massive Sgs-glue protein secretion would have taken place much later in their evolution.

This issue deserves more attention in the future. With currently available data, we do not have a reason to think that, during evolution, the loss of Sgs-glue genes led to a complete disappearance of glue secretion. If this were to have occurred, the reappearance of Sgs-glue secretion several times during dipteran evolution would seem to require new sets of glue genes to evolve and these would have low or no similarity to the currently known Sgs-genes/proteins. However, if regulatory changes at the two or three phylogenetically oldest *Sgs*-genes (*Sgs3* and *Sgs5*/*Sgs5bis*) were to lead to alterations in their expression, Sgs-glue secretion could be lost in practical terms. Following novel environmental changes, the ancestral genes could serve repeatedly for gene duplication and the evolution of new *Sgs*-genes. Alternatively, but also very speculatively, loss of some glue genes could have occurred if they were recruited for another non-glue function during evolution, at least in some species. Unfortunately, we cannot clearly answer the question of whether the nine species lacking glue secretion are phylogenetically clustered. Only three (*D*. *willistoni*, *D*. *albomicans*, and *D*. *mauritiana*) of them have been sequenced, and we have phylogenetic comparison of *Sgs*-genes in only two species that are phylogenetically distant (*D*. *willistoni* and *D*. *mauritiana*). More fully understanding the larval life-history of all of these species and placing their natural developmental and breeding environment in the context of the complex set of prepuparial events (Sgs-synthesis, Sgs-secretion, glue expectoration, gluing to the substrate, place of pupariation) should provide an important link enabling the identification of relationships to assemble a clearer evolutionary picture of the evolution of Sgs-glue secretion.

A possible explanation for the absence of Sgs-glue production in some species is that it has been suppressed epigenetically by the rearing conditions. In our view, however, this seems unlikely. Expression of Sgs-proteins is known to be under the strict control of an ecdysone-regulated developmental program, as is the process of Sgs-glue exocytosis. Ecdysone as a strong developmental/temporal factor acts *via* the elaboration of a transcriptional cascade that we suspect has the potential to overcome many epigenetic mechanisms, especially those that would originate in environmental cues (Kress and Swida 1990; Karim et al., 1993; von Kalm et al., 1994; Lehmann, 1996; King-Jones and Thummel, 2005; Farkaš et al., 2011). In addition, the same ecdysone cascade itself utilizes also epigenetic factors. It is unclear how epigenetic factors would be able to selectively target only the complex set of events leading to Sgs-glue expression and exocytotic secretion, while not also preventing other phases of this developmental program or even the entire program.

### An increase in SG cell number also provides for increased apocrine secretion that enhances antimicrobial defense

Inferences about the relationship between the number of secretory cells per lobe and the production and secretion of an Sgs-glue are valid only for the *Drosophilidae*, however. It is striking that the representative of *Calliphoridae* we evaluated, *L. cuprina*, which does not need to fix its puparium to a substrate and does not produce any Sgs-glue, and which may not have any *Sgs*-genes, has 437 to 448 secretory cells per lobe. This is three-to nine-fold more than the number in *Drosophila* species, whether or not the species exocytotically releases an *Sgs*-glue (*D*. *melanogaster*: 3.3-fold, 134 cells/lobe; *D*. *simulans*: 3.6-fold, 123 cells/lobe; *D*. *yakuba*: 4-fold, 111 cells/lobe; *D*. *atripex*: 4.3-fold, 104 cells/lobe; *D*. *parabipectinata*: 4.6-fold, 98 cells/lobe; *D*. *funebris*: 4.7-fold, 96 cells/lobe; *D*. *montana*: 5.0-fold, 89 cells/lobe; *D*. *bipectinata*: 5.5-fold, 82 cells/lobe; *D*. *pseudoobscura*: 6.5-fold, 69 cells/lobe; *C*. *costata*: 9.3-fold, 49 cells/lobe). This suggests an altogether different conclusion that is pertinent to the function of apocrine secretion in the prepupal SGs. Since *Drosophila* species with higher numbers of secretory cells such us *D*. *melanogaster*, *D*. *simulans*, and *D*. *yakuba* show a more profound apocrine secretion during their prepupal period, there appears to be a relationship between the number of secretory cells and secretory activity, even if it is not *via* exocytosis but by an apocrine mechanism. In this case, the very high number of cells per lobe in *L. cuprina* (and also in *H. illucens* having 158 to 170 cells per lobe) may be associated with a long lasting or perhaps a continuous, albeit low-level of secretory activity that reflects another environmentally related function. The larvae of *L*. *cuprina* and *H*. *illucens* live in a strongly decomposing and suppurating environment, and a more persistent production of antimicrobial peptides by a higher number of cells, possibly released to their alimentary tract by a steady low level of apocrine secretion during all of the larval instars, may reflect a specific adaptation to this habitat. This may even explain why the larvae of *L*. *cuprina* are successfully used in maggot debridement therapy. Thus, there is a simple explanation for the apparent paradox that a higher number of SG cells per lobe is related to a greater production of Sgs-glue, and more intense exocytosis in larvae on one side as well as a more profound, later apocrine secretion on other side. This is the use (evolutionary co-option) of the same set of secretory cells for two related developmental functions. While their large number may be evolutionarily tuned for merocrine purposes, it also provides for a massive and more intense non-vesicular protein release (apocrine secretion).

### Apocrine secretion is an evolutionarily ancient mechanism

Another crucial inference is related to the evolutionary age of the fly species we scrutinized. While apocrine secretion is present at some level in all of the *Drosophilidae* species we examined, it is more intense in the evolutionarily younger *D*. *melanogaster*, *D*. *sechellia*, *D*. *mauritiana*, *D*. *simulans* than evolutionarily older species groups such as *D*. *pseudoananassae*, *D*. *willistoni*, *D*. *bipectinata*, *D*. *pseudoobscura*, *D*. *persimilis*, *D*. *mojavensis* species of the *melanogaster* subgroup. It is also present in the evolutionary distant and older species *L*. *cuprina* and *H*. *illucens*. *H*. *illucens* is representative of the family *Stratiomyidae* (*Stratiomyomorpha*) which is as old as 185–210 million years (Hennig, 1981; Beverly and Wilson, 1984; Friedrich and Tautz, 1997; Grimaldi and Cumming, 1999; Woodley, 2001; Yeates and Wiegmann, 2005). Therefore, the apocrine function of salivary glands to produce exuvial and immunoprotective fluid could be conserved from very early in the evolution of the *Diptera*.

**FIGURE 6:**
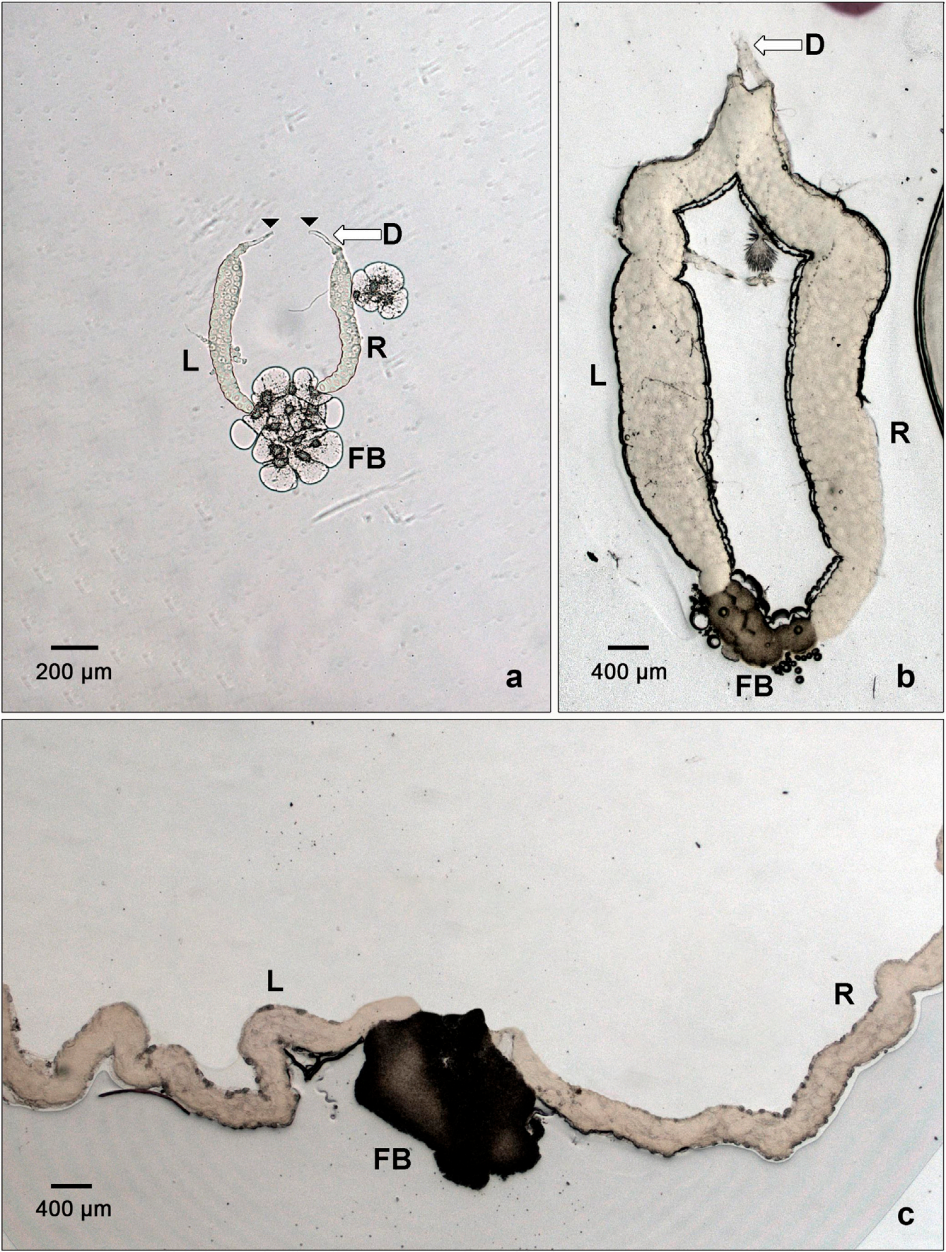
The SGs of *C. costata*, *L. cuprina*, and *H. illucens* show accretion or coalescence at their posterior ends. In all insects, the anterior ends of the SGs typically fuse together to form a common duct. However, it is very rare to see their posterior ends in proximity to each other. This only has been observed in a few phylogenetically older groups of insects, including some dipteran species. In C. *costata*, the single representative of *Drosophilidae* among the three species shown here, the posterior ends of the salivary glands appear to be embedded in a broader fat body (FB) area having large fatty cells **(A)**; in this preparation the anterior ducts have been separated and freed (arrowhead). In the maggot fly, *L. cuprina*, the posterior ends of the significantly larger SGs are glued into a much darker fat body (FB) mass **(B)**. The ducts (D) of these glands are much shorter and thicker than in other dipteran species and are not easily separable. **(C)** illustrates that the SGs of *H. illlucens* are at least 5-times longer than those of *L. cuprina*. Consequently, their anterior ends are out of the imageable area even at lowest usable magnification (lense 2.5×). Their posterior ends are impaled into a dark mass that appears to be a fat body (FB). L, left gland lobe; R, right gland lobe. The scale bars in the lower left corners represent 200 or 400 microns.

In the course of this work, we made an intriguing anatomical observation concerning the salivary glands of *L. cuprina*, *H. illucens*, and *C. costata*. In these species, the posterior ends of the SGs touch each other, being impaled in a common shred of adipose tissue resembling a knot-like bundle or coalesced accretion (Figure 6). This structure is most prominent in *L*. *cuprina* and *H*. *illucens*, and less in *C*. *costata*. This raises the question whether the evolution of *Sgs*-genes and production of Sgs-glue could impact the separation of the posterior ends of SGs lobes and be associated with the loss of their contact with the posterior fat body. This anatomical arrangement of SGs is more frequently observed in evolutionary older groups (Keilin, 1915; Keilin, 1917; Oldroyd, 1964; Yeates and Wiegmann, 2005), though we are unaware of a functional explanation. This will require further investigation.

### Sgs-glue secretions are not required for pupariation outside of food

Our research has also uncovered another intriguing relationship between the preferred site of pupariation and the presence (or absence) of the Sgs-secretory granules in the larval SGs: some species that do not form typical Sgs-granules are still capable of pupariating outside of the food (on the wall of a glass vial or a piece of folded filter paper), while some species that do produce Sgs-granules and even secrete them by exocytosis still pupariate in the food. These Sgs-producing and in-food pupariating species mostly have longer anterior spiracles which almost always protrude from the food. Importantly, this suggests that these species do not need to attach to a substrate that is a long distance from the food source. This may be a species-specific adaptation to an environment (original natural habitat) with poor food resources and may provide for easier access of the eclosed adults to the food that is required for breeding. Nevertheless, their food still contains a high load of various, potentially pathogenic microorganisms. Since the Sgs-secretion is released by a pupariating larva and circumferentially embraces a large portion of the outer puparium surface, the Sgs-secretion has evolved a dual function: affixing the puparia to a substrate and providing a physical encasement for antimicrobial protection. This possibility was recently suggested by us (Beňová-Liszeková et al., 2019) based on findings that the apocrine secretion contains robust antimicrobial properties; it finds additional support here.

How then do species not producing any or only a small Sgs-secretion attach to a substrate? The answer is unclear when the substrate is the wall of a glass or plastic vial. However, a mechanism became clear when pieces of filter paper were added to the culture for this purpose prior to pupariation: the wandering larvae chew up and ruminate the filter paper using their mouth armature to liberate 200–1,500 μm long cellulose fibers that subsequently become attached in an irregular manner to the surface of their cuticle (puparial case), thus firmly attaching them to the paper substrate.

It appears that the anterior spiracles of some species of *Drosophila* (*D*. *hydei*, *D*. *funebris*, *D*. *albomicans*, *D*. *sulfurigaster*, *D*. *immigrans*, and *D*. *robusta*) are not functional in the larva when they are immersed deep in the food mass, becoming capable of admitting air only in the prepupa and pupa. Before forming a puparium, these species’ larvae crawl to a position which is relatively dry (or at least distant from other larvae) where they can be assured of securing air. Usually their entire puparium is exposed to air. Especially in a crowded culture, however, some pupae may be situated beneath the food, so that only the end of the anterior spiracle is exposed to air. As described in Robertson’s classic work (Robertson, 1936), puparia having only their anterior end exposed to air develop and emerge normally. Practically, it seems, therefore, that they must have obtained all their pupal-period oxygen using their anterior spiracles. As Robertson (1936) noticed, if a puparium is formed entirely beneath the food, the individual does not develop very far, probably because it becomes asphyxiated. Among the above-listed six species which come from at least three different continents and various habitats, three are known to not have any visible Sgs-granules or glue secretion (*D*. *albomicans*, *D*. *immigrans*, and *D*. *robusta*), and the only species that pupariate regularly in the food (*D*. *albomicans*), produces very little Sgs-glue. Therefore, it is tempting to conclude that an absence of Sgs-granules is not directly linked to the preferred pupariation in the food, and that longer anterior spiracles, which could facilitate breathing while embedded in such an environment is not directly or exclusively linked to pupariation within food.

Indeed, it is difficult to find clear-cut correlations between pupariation behavior, the presence of collector sacculae, or the presence of post-apocrine concrements with clusters of species based on their climatic or geographical region, phylogenetic relationships, or SG-cell number. When a set of similar attributes are present in a group of species, at least one species is from a different climatic or geographical region, has a more distant phylogenetic relationship, or has a completely different SG-cell number. How SG-cell number, phylogeny or climatic region contributes is elusive not only to us but to generations of other drosophilists. For the few species that are described in the literature (*D*. *melanogaster*, *D*. *ananassae*, *D*. *virilis*, and *D*. *hydei*), our observations are similar to those of previous authors (Vandal and Shivanna, 2007; Vandal et al., 2008). They reported that these species pupariate on a glass wall, whereas *D*. *bipectinata* preferred to pupariate on the fruit which was present in the food. Erezyilmaz and Stern (2013) confirmed that species as *D*. *sechellia* prefer to pupariate within their host fruit, i.e., within their food. Unfortunately, we did not include several other species (*D*. *malerkotliana*, *D*. *rajasekari*, *D*. *rubida*, *D*. *nasuta*, and *D*. *pararubida*) in our study where information has been reported for the site of pupariation (Shivanna et al., 1996; Vandal et al., 2008) and other aspects of pupariation such as conspecific aggregation and/or the height of the pupariation site (Singh and Pandey, 1993; Beltrami et al., 2010). One important and possibly critical factor missing in many studies on non-*melanogaster* species is the natural preference of larvae for food during their development. For the majority of species, there is mostly only knowledge about the natural distribution and seasonal occurrence of adults and very little if any data are available about larval life [cf., Grossfield, 1971; Throckmorton, 1975; Lachaise and Tsacas, 1983; Ashburner, 1989; Markow and O’Grady, (2005)]. This missing piece of the mosaic could be an important link to identify a unified or shared denominator linking these observations on pupariation behavior, collector sacculae, and post-apocrine concrements among otherwise unrelated species. In a few cases, it is clear that evolutionarily or geographically distant species can have some consimilar food and breeding adaptations.

### Pre-apocrine sacculae and post-apocrine concrements likely represent evolutionary specializations to enhance the antimicrobial functions of exuvial fluid

We observed a previously uncharacterized structure in the prepupal SG of seven species that we named pre-apocrine sacculae. The structure appears to serve as a collector organelle where the secretory material is gradually concentrated at specific foci for the future secretion. These sacculae become visible about 2 h prior to when their contents are abruptly released into the SG lumen. Laser confocal microscopy revealed that the fluorescent signal of two and sometimes all three of the exemplar proteins we studied becomes completely missing from their original subcellular locations as it becomes localized to sacculae during this period. This indicates that an active process is used to recruit, transport, and deposit cytoskeletal, membrane, cytosolic or nuclear proteins within these organelles for subsequent secretion. We propose that the sacculae serve as a unique interstep between recruitment, transport and secretion of the protein mass. Most probably they represent a specific progressive adaptation that provides for the more efficient release of apocrine material so that the luminal secretion can become instantly available. Quick delivery of the exuvial fluid to the periexuvial space would be especially advantageous if the process of pupation is very fast, which is what we observed in the species with sacculae. There are several compelling questions concerning these structures: what are they composed of, do they have a membranous or vesicular character, how do they facilitate the emptying of their contents into the SG lumen, and how is such an abrupt release of their contents into the lumen and periexuvial space achieved? It cannot be exocytosis as we know it as thus would take several hours, if it were similar to Sgs-glue secretion in larvae (Lane et al., 1972; von Gaudecker, 1972; Boyd and Ashburner, 1977; Farkaš and Šuťáková, 1998). We speculate that the rapid release of secretory material may be accomplished by the generation of a type of turgor-like intracellular pressure, or by the peristaltic waves of extracardiac hemolymph pressure that are known to occur during pupariation and pupation of many insects, including flies (Sláma, 1976; Sláma, 1984; Sláma, 2000; Žďárek et al., 1979; Žďárek, 1985). We are currently characterizing these novel organelles using transmission electron microscopical analysis to obtain a more clear-cut understanding of their physical structure.

We also discovered the presence of novel post-apocrine concrements inside the SG lumen of eight species (*D*. *willistoni*, *D*. *mojavensis*, *D*. *subobscura*, *D*. *albomicans*, *D*. *eugracilis*, *D*. *sulfurigaster*, *D*. *equinoxialis*, *D*. *immigrans*). Interestingly, five of these eight species (*D*. *sulfurigaster*, *D*. *equinoxialis*, *D*. *immigrans*, *D*. *albomicans*, and *D*. *eugracilis*) share both pre-apocrine sacculae (collector organelles) and post-apocrine concrements. All these species come either from Southeast Asian or Australian ecozones, so the pre-apocrine sacculae and the post-apocrine concrements may either share a common ancestral origin, and/or be causally/functionally related. Fundamental questions concerning them are what they represent—how do they arise, and what function do they serve? By the time when these concrements are found in the SG lumen, no proteins can be detected by the antibodies we used. We previously observed that in the very late prepupal SGs of *D*. *melanogaster* 1–2 h following apocrine secretion, there is a massive excretion of calcium oxalate (CaOx) that reflects active nephridial-like anion transport (Farkaš et al., 2016). Based on the timing of their appearance following apocrine secretion, we suggest that the concrements found in these eight species may arise from an analogous type of excretory activity. Since our initial report of the oxalates in *D*. *melanogaster*, we found that there are also present additional organic salts (bicarbonates, fumarates, butyrates, malates, pyruvates, valerates, citrates, succinates, acetates etc., Farkaš et al., unpublished data) that most probably contribute to the soup that is the exuvial fluid. As exemplified by CaOx, these organic compounds provide additional germicidal substances. In contrast to how CaOx leads to insoluble urinary stones in many mammals, it remains soluble in the exuvial fluid of *D*. *melanogaster*. This facilitates its distribution and delivery into the very narrow periexuvial space. However, no concrements or “incrustations” can be observed in the lumens of prepupal SG of *D*. *melanogaster*. Hence, if post-apocrine concrements in these eight species are associated with excretory activity like that seen in *D*. *melanogaster*, it will be important to address what mechanism generates concrements that appear to be insoluble and how they are delivered to the periexuvial space.

Based on their color, one could speculate that the post-apocrine concrements contain melanin or melanin-like substances. It is well established, however, that melanin formation, except for tanning of the cuticle and cuticular structures (True et al., 1999; Minakhina and Steward, 2006), is associated with non-specific defense reactions against foreign particles, parasites, and microorganisms (Eleftherianos and Revenis, 2011; Nam et al., 2012; Schneider and Imler, 2021; Evans et al., 2022; Liegeois and Ferrandon, 2022). The formation of the melanin is a reaction activated by a direct physical challenge from a parasite or microorganism (e.g., their surface antigens). Except in mutant animals, exemplified by the *catecholamines-up* (*Catsup*) gene (Stathakis et al., 1999), the process of melanization is not known to start freely or automatically without such a stimulus. Indeed, in *D. melanogaster*, where concrements are not found, FlyAtlas and modENCODE transcriptomic data indicate that expression of tyrosine hydroxylase and dopa decarboxylase, two enzymes which function in the formation of melanin, is very low in the larval and prepupal salivary glands, comparable to illegitimate expression (Chintapalli et al., 2007; Roy et al., 2010; Graveley et al., 2011; Robinson et al., 2013; Chen et al., 2014). Certainly, the composition and mechanism of the formation of dark brown concrements deserves further attention.

### Why is BR-C detected in the very late prepupal, but not the larval, SGs of two non-*Drosophila* flies?

In contrast to all *Drosophila* species (DiBello et al., 1991; Emery et al., 1994; von Kalm et al., 1994; Crossgrove et al., 1996; Bayer et al., 1997; Mugat et al., 2000) and even some other non-dipteran holometabolous and even hemimetabolous insects (Zhou et al., 1998; Erezyilmaz et al., 2006), the ecdysone-regulated transcription factor BR-C was never detected in the larval SGs of two non-*Drosophila* flies (*C. costata* and *L. cuprina*). Surprisingly, however, it was detected only in the very late prepupal SGs during the release of the apocrine secretion into the SG lumen. This suggests that in these two rare cases, BR-C is not required for initiation of metamorphosis (pupariation) and early prepupal stages. This is unlike other *Diptera* and holometabolous insects and is in striking contrast to our current knowledge on the function of this gene (Emery et al., 1994; Bayer et al., 1997; Erezyilmaz et al., 2006; Zeng and Hou, 2012). Our data indicate that BR-C is never detected in nuclei, its expected subcellular location, but that in these two species it must be synthesized just prior to the apocrine phase. They suggest that nascently synthesized BR-C protein is prevented from entering nuclei and instead is immediately recruited to the apocrine transport machinery. BR-C as transcription and chromatin remodeling factor is required for controlling ecdysone-regulated gene expression at the onset of metamorphosis and known to act exclusively only in nuclei (Karim et al., 1993; Emery et al., 1994; Kuchárová-Mahmood et al., 2000; Farkaš et al., 2011). It is essential for future research to confirm that these observations do not reflect the masking of BR-C from detection by the antibodies we used and that it is not produced in *C. costata* and *L. cuprina* during any previous stage to serve its expected nuclear function, why it is produced just prior to apocrine secretion, and as such, why it is required in the secretory material and exuvial fluid?

### It will be insightful to investigate additional species

Finally, it would be immensely valuable to complete a set of observations on *D*. *gibberosa*, as it is one of the larger neotropical species of *Drosophila* that is known to pupariate preferentially inside food, and its SGs show maximum secretory output in the prepupa rather than in the late third-instar larva (Roberts and MacPhail, 1985; Roberts, 1988; Shirk et al., 1988; Tosi et al., 2007). These characteristics suggest that observations on this species, which we could not obtain during the last several years from the fruitfly community, would test the prediction that its SGs are oriented towards apocrine secretion rather than towards the production of Sgs-glue, and so provide enlightenment as to the relationships between SG function, cell number, secretory capacity, and even larval prepupariation behavior.

## Data Availability

The original contributions presented in the study are included in the article/supplementary material, further inquiries can be directed to the corresponding author.
